# Why is William Sharp's name forgotten when his novel method for treating fractures of the Ankle is still used today?

**DOI:** 10.1177/09677720221082103

**Published:** 2022-03-23

**Authors:** Sean P Hughes, G Anne Davies

**Affiliations:** Surgery and Cancer, 4615 Imperial College London, London, UK

**Keywords:** William Sharp, Granville Sharp. Johan Zoffany, Guillaume von Dupurtren, Fracture /Dislocations of Ankle, Percivall Pott

## Abstract

In 1837 Guillaume von Dupuytren (1777–1835) wrote that the innovative method of reducing an ankle fracture by relaxing the calf muscles was due to both William Sharp (1729–1810) and Percivall Pott (1714–1788). While history records the many surgical achievements of Percivall Pott, little is known of William Sharp's contribution. He is probably best known as one of a remarkable family portrayed by Johan Zoffany (1733–1810) and exhibited at the Royal Academy in 1781. We review William Sharp's career and contribution as a surgeon to the treatment of fracture/dislocations of the ankle and ask why his concept is not better known today.

## Introduction

In 1837 the French surgeon Baron Guillaume von Dupuytren (1777–1835) in *On the Injuries and Diseases of Bones* about fractures of the ankle joint commented

To William Sharp and Percival Pott belongs the honour of having established that the fundamental condition necessary to the reduction and maintenance of a fracture in a proper position is, that the muscles should be as much relaxed as possible… The advantages of this plan, which were probably exaggerated by Pott, though too lightly treated by Desault, were so evident, that it was speedily adopted and became popular in France.^1^

Before this observation that the muscles of the calf need to be relaxed in order to reduce a fracture/dislocation of the ankle joint, these injuries were managed by traction as taught by Galen (129-c210 AD). Galen advised that the fractured bones should be realigned by strong extension followed by direct compression to the joint in order to reduce the dislocation.^2^ While history records the many surgical achievements of Percivall Pott (1714–1788) and his approach to reducing ankle fractures by relaxing the calf muscles, little is known of William Sharp’s contribution to this novel idea or why Dupuytren referred to him.

**Figure 1.**
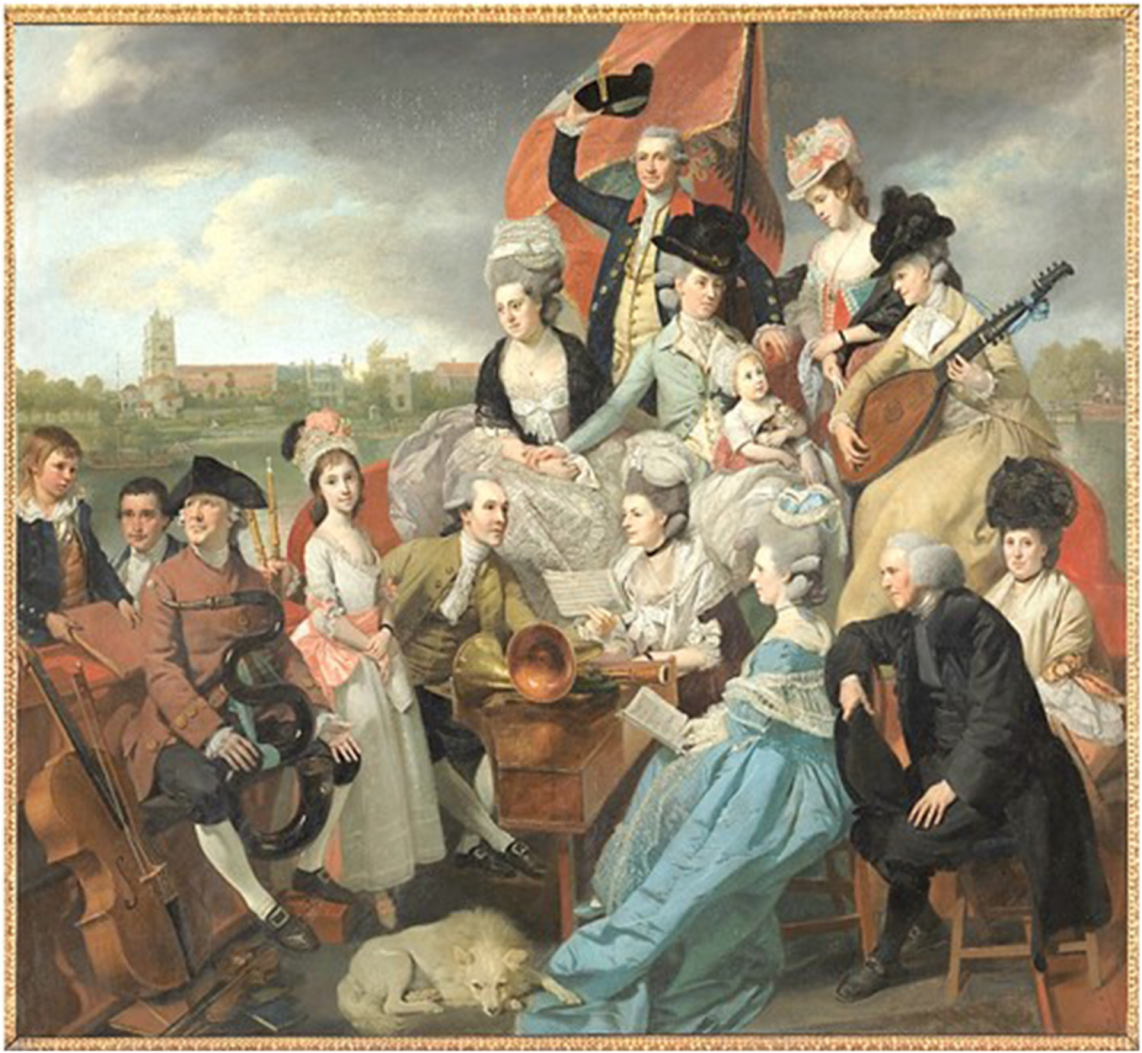
Sharp family (1779-1781) by Johan Zoffany, National Gallery London, © Lloyd-Baker estate. William is at the back waving his hat, with his left hand on the tiller.

Indeed, if William Sharp, is known to us today it is probably because of the portrait of the Sharp family by Johan Zoffany (1733–1810) which hangs in the National Gallery in London (Figure 1).

William commissioned the portrait which shows his brothers and sisters, and their children along with their musical instruments aboard his barge ‘*The Apollo’* on the River Thames. In addition to the painting, which alone would ensure a lasting record, William might also be known as the brother of the abolitionist Granville Sharp (1735–1813). Less well-known than William Wilberforce (1759–1833) and Thomas Clarkson (1760–1846), Granville was the instigator of repeated legal challenges that culminated in the case of James Somerset and the landmark Mansfield judgement on slavery in 1772 which ruled that in England, no master was allowed to take a slave by force to be sold abroad.^3^ In Northumbria, where William Sharp was born and grew up, he is more likely to be known as the brother of John Sharp (1722–1792), Archdeacon of Northumberland in the Diocese of Durham and Trustee of the Lord Crewe Charity which pioneered welfare services from its base in Bamburgh Castle.

The fact that William Sharp commissioned the family portrait suggests that his intention was to ensure a lasting memorial, perhaps a personal rather than a surgical legacy, not only for himself but for his siblings and the whole Sharp family. His wish was evidently to be remembered for the music making *en famille*, on the river, rather than in his chosen profession as a surgeon. Also, that he elected to be depicted in the Windsor Uniform suggests that he wanted to be associated with Royal favour and patronage. Indeed, George III did call on William's services as a surgeon, though his was never a Royal Appointment,^4^ and this makes it all the more interesting that William's contribution to surgical knowledge has remained almost unrecognised.

In this paper we explore William Sharp’s background and career and review his contribution as a surgeon to the treatment of fracture/dislocations of the ankle. We ask why his novel idea for the management of these severe injuries is not better known today. Could the reputation of Percivall Pott, his contemporary at St Bartholomew's Hospital in London, have been enhanced at the expense of William Sharp's? Was it because Pott not only taught but also wrote extensively on many aspects of surgery that he is remembered? The answer would appear to satisfy the law of eponymy, proposed by the American economist SM Stigler in 1980, that no scientific discovery is named after its original discoverer.^5^

We would argue that William Sharp was a practitioner rather than an educator and gave little thought to promoting himself or advertising his new method of treatment. The evidence suggests that he had no difficulty in achieving what today is referred to as a ‘work-life balance;’ his family life and musical performance would not be sacrificed in the interests of his career in surgery.

**Figure 2.**
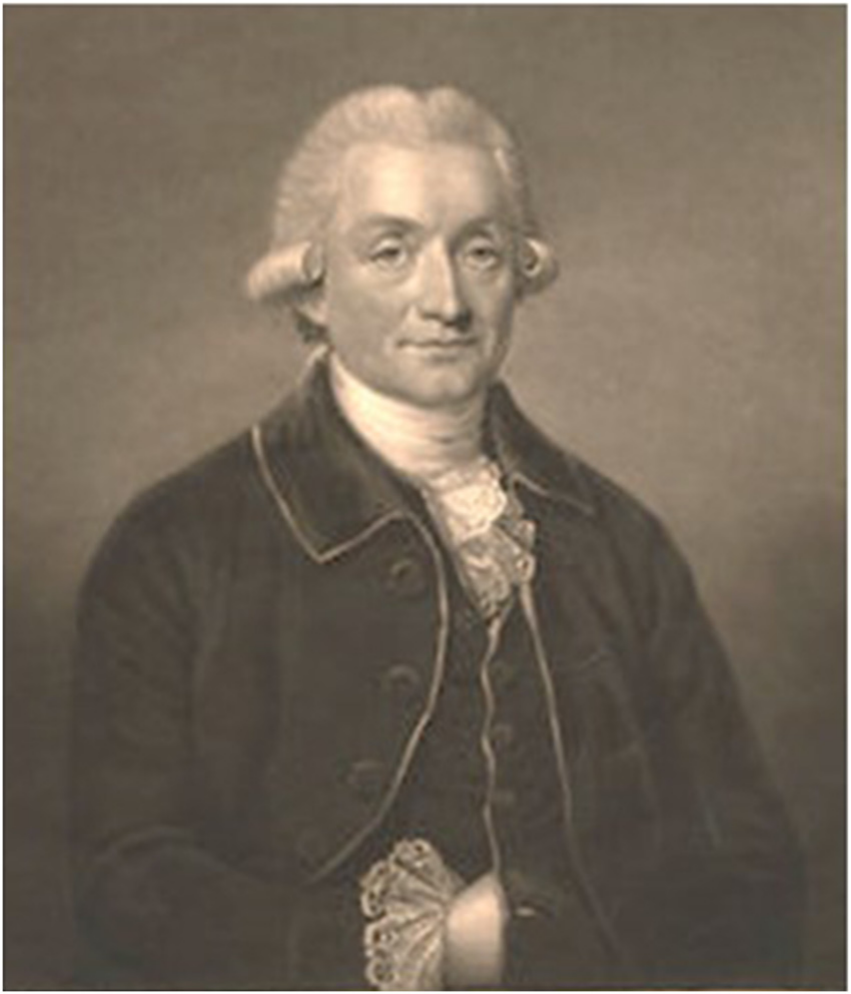
William Sharp engraving by Charles Turner from a portrait by J Abbot 1794 © National Portrait Gallery, London.

**William Sharp** (Figure 2)

William Sharp was born in 1729 at Whitton Tower, then the Rectory, at Rothbury in Northumberland, where his father, Thomas Sharp (1693–1758) a Doctor of Divinity was the Rector and Archdeacon of Northumberland, in the Diocese of Durham. His grandfather, John Sharp (1645–1714), had been Archbishop of York. William's mother, Judith was the youngest daughter of Sir George Wheler (1765–1723) a wealthy landowner, distinguished botanist, and travel writer from Charing in Kent, who was also ordained and was a prebendary at Durham Cathedral.

William was one of nine children who survived infancy. An older brother Charles died in his mid-teens. The two eldest boys, John (1723–1792) and Thomas (1725–1772), attended Durham Grammar School (now Durham School) and went to Trinity College, Cambridge, their father's old college, both becoming clergymen. James (1731–1783) and Granville (1735- 1813) were younger than William and there were three sisters; Elizabeth, (1733–1810) Judith, (1733–1809) and Frances (1738–1799). The lifelong closeness of the relationship between William and all his siblings testifies to their happy childhood and the care with which they were brought up. ‘Two such happy homes we had been blessed with’, is how Elizabeth recorded in her diary their upbringing in the Rectory at Rothbury and her father's prebendal house in the College at Durham.^6^

In 1743 at the age of fourteen his father arranged for William to be apprenticed to James Phillips, a surgeon at St Bartholomew's Hospital in London. The apprenticeship was completed by 1750 and, on retirement, Phillips made over his house in Mincing Lane to William who set about building up a practice and ran a surgery there, possibly for the poor. In 1755 he was elected as an Assistant Surgeon to St Bartholomew's Hospital:

The Court then proceeded to the Election of an Assistant Surgeon of the Hospital, in the Room of Stafford Crane, chosen to be one of the Surgeons of the Hospital. And the petitions of Edmund Pitts, Edward Roberts, John Ruding, William Sharp, Richard Webb and William Williams, Surgeons, were Read, severally praying to be chosen into the said Vacant place. And the said Edmund Pitts, Edward Roberts, John Ruding, Richard Webb, and William Williams having declined the Ballot [sic], the said William Sharp was unanimously Elected. It is thereupon thought Fitt and Ordered that the said William Sharp shall be one of the Assistant Surgeons of this Hospital during the Governors’ pleasures.^7^

In 1765, aged 36 William Sharp married Catherine Barwick, **(**1741**–**1814) who had many miscarriages before giving birth in 1778 to Mary, their only child. In 1769 they moved to 8 Old Jewry, a much larger house and William continued to combine his work at St Bartholomew's Hospital with his evidently lucrative practice. He was an early advocate for smallpox variolation, still relatively new at that time and not without risk. His sister Elizabeth recorded her own inoculation by him, and James's daughter Catherine (Kitty) underwent variolation as a small child in 1771, with her Aunts Elizabeth and Frances hastening to London from holiday in Durham to be on hand should the child have an adverse reaction.^8^ William's commitment to the prevention of smallpox by inoculation led to a steady demand for his services. Hester Thrale (1741–1821) a close confidante of Samuel Johnson (1709–1784) recorded in her diary for 15 December 1780

This day I inoculated Cecilia Margaretta Thrale, and Henrietta Sophia Thrale her youngest sister: god send us safe thro! Sharp is the Operator; he won my heart by his Tenderness & Skill and Assiduity about my master's Carbuncle: and it is so comfortable to have regular People about one.^9^

Hester Thrale was extremely well connected, and her praise a recommendation worth having. Her writing gives us a rare insight into William Sharp as a kindly and skilled medical practitioner.

Having established a reputation and built up his practice, William Sharp resigned from St Bartholomew's Hospital on 1 December 1778:

Wherein he desires to Resign his Office of Assistant Surgeon to this Hospital being unable from the Engagements of his own Private Business to give that Attendance he thought his Duty to the Hospital required.^10^

Following tradition, Sharp donated £50 to the hospital and was nominated by the Treasurer to become a Governor. A month later his brother James, who by now was a successful iron merchant in Leadenhall Street and had been elected to the Common Council of the City of London, joined William as a Governor of the hospital.^11^

**William Sharp and Fractures of the Ankle**

On 12 February 1767, Sharp wrote a letter to Dr James Parsons (1705–1770), Assistant Foreign Corresponding Secretary to the Royal Society of London, which was published in the Philosophical Transactions of the Royal Society:

When a surgeon is called to a fractured leg, at the place where the accident happened, let him lay the patient on the injured side, upon a flat surface; and raise the knee of the fractured limb towards the abdomen, bending at the same time the knee joint, so as to put the extensor muscles of the foot (which are the strongest) into a state of relaxation. He will then be enabled to replace the ends of the fractured bones, and restore them to the proper situation, without the customary strong extension of the limb, which is troublesome to the surgeon, painful to the patient, and apt to bring on tension, spasms, and inflammation of the muscles.^12^

This observation was new, appearing before Pott had written about this in his textbooks. It would seem therefore that William Sharp developed his own views on ankle fractures and their treatment independently of Percivall Pott.

In his letter Sharp went on to describe a method for holding the fracture in place, which consisted of splints made of strong pasteboard reinforced with glue and placed around the injured leg, held in position by a many tailed bandage. In the letter Sharp emphasised the importance of flexing the knee joint in order to let the muscles of the calf relax so that the fracture/dislocations would reduce.^13^

Mainly on the basis of this work, in 1769, Sharp was elected a Fellow of the Royal Society of London, the citation stating

Mr William Sharpe^14^ of Mincing Lane, One of the Surgeons of St Bartholomew's Hospital. A gentleman very Eminent in his profession, and well versed in Other parts of Natural Knowledge, and the happy inventor of a truely Mechanical Instrument for Securing fractured legs with great Ease and convenience to the Patient, (an account of which is printed in the Last volume of our Transactions) being very desirous of the Honr of being elected a member of the Royal Society: we the undernamed do, from our personal Knowledge, recommend him as one that will prove a very useful member thereof, and highly deserving the Honor.^15^

One of the signatories to Sharp's application was Percivall Pott.

**Percivall Pott and Fractures of the Ankle**

Percivall Pott, a renowned surgeon, teacher and writer was born in 1714 in Threadneedle Street London. He was the son of a draftsman who died when Percivall was three years old. His education was funded by Francis Atterbury (1663–1732), Anglican Bishop of Rochester who was related to Pott's mother. Atterbury later financed Pott's apprenticeship to Edward Nourse (1701–1761), an Assistant Surgeon at St Bartholomew's Hospital. After completing his apprenticeship Pott moved to Fenchurch Street and continued working at the Hospital. At a meeting of the General Court of St Bartholomew's Hospital London 30 November 1744, Pott was appointed to succeed James Phillips as Assistant Surgeon, becoming a full surgeon in 1749.^16^ He continued with his practice in Princess Street, Hanover Square, and wrote three volumes of surgical treatment which included the management of fracture/dislocations of the ankle. His interests though went far beyond the treatment of fractures. He also published work on scrotal cancer in chimney sweeps, tuberculosis of the spine, the analysis of muscle palsy, fistula-in-ano and diseases of the eye.^17^

James Earle wrote in his account of Percivall Pott

The observations and instructions which thus flowed from his ready pen, were enforced by his practice, and illustrated by oral communication: and he was happy to embrace every opportunity which his situation gave him, of conveying the information he had collected to those who had not the same means of acquiring it.^18^

Pott later received widespread recognition by his peers. He was elected Master of the Company of Surgeons in 1765, the forerunner of the Royal College of Surgeons of England and became the first Honorary Fellow of the Royal College of Surgeons of Edinburgh in 1786. On retirement in 1787, he was elected a Governor of St Bartholomew's Hospital as in due course William Sharp would be. On his election to the Royal Society of London in 1764, Pott's citation made specific reference to his publications, recording: *‘*he was a gentleman very well known to the learned world by several treatises he has published.’^19^

**Figure 3.**
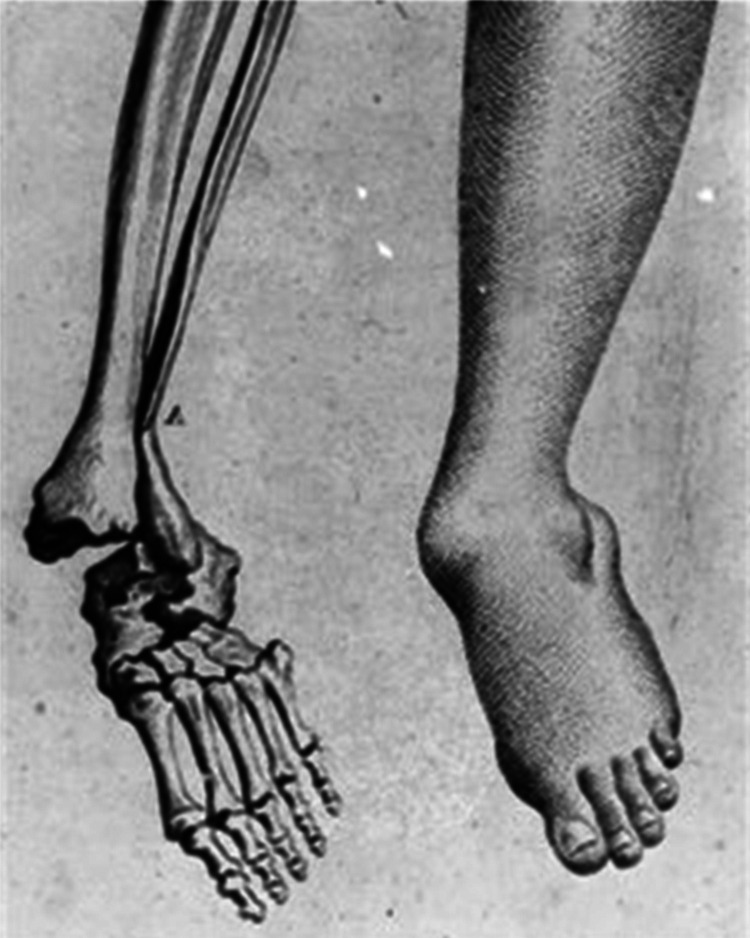
Pott’s drawings of a fracture dislocation of the ankle where both the medial and lateral malleoli are fractured, and the talus is no longer within the ankle mortise. *The Chirurgical Works of Percivall Pott*, facing p 313, figure M. (20).

In *Fractures and Dislocations* published in 1769. Pott discussed in detail the management of fracture/dislocations of the ankle.^20^ (Figure 3)

Pott described a new technique for managing fracture/dislocations of the ankle, writing: *‘*Put the limb into such a position as shall relax the whole set of muscles belonging to or in connection with the broken bone.^21^

He then discussed the action of muscles on a fracture and the fact that traction in extension can injure the muscles resulting in their inflammation, as had been noted by Sharp. Pott argues that muscles which are stretched in extension, prevent reduction and that the muscles need to be relaxed to achieve reduction of the dislocation:

Is it not obvious, that putting a limb into such position as shall relax the whole set of muscles belonging to or in connection with the broken bones must better answer such purpose?^22^

Pott, as did Sharp, stressed the need to bend the knee in order to relax the muscles of the lower limb and explained that this technique was practised at St Bartholomew’s Hospital.^23^ In this book there is a drawing of the flexed knee which is similar to that of Sharp's.^24^

Pott later referred briefly to Sharp but only to his splints:

If Mr Sharpe's splints be made use of, there is in one of them a provision for the easier support of the foot and ankle, by an excavation in, and a prolongation of the lower or fibular splint, for the purpose of keeping the foot steady*.*^25^

Pott makes no other mention of William Sharp and there is no discussion of Sharp's ideas on the relaxing muscles in order to reduce a fracture. Later, other surgeons took up Potts’s description of treating fracture/dislocations of the ankle, including Charles Bell (1774–1842) who supported Potts new method in that *‘*in general the joint is to be relaxed and the limb placed in half bent position’.^26^ Bell disliked the traditional Galenic method of extension and traction, which was used to reduce a fracture/dislocation, particularly if it required the use of mechanical devices:

Patients were fitted with engines not unworthy of the chambers of the Inquisition … Have we not machines and belts innumerable… Have we not since the ancient Greeks had Windlass and Ship block, the invention of Archimedes himself dignified with title of Glossocoma [a hoisting winch].^27^

In his long and successful career Pott was not always free from criticism. In 1756 he was accused of plagiarism by William Hunter (1718–1783) anatomist and physician. Hunter alleged that Pott had stolen from him and his brother John Hunter (1728–1793) ‘The Knowledge and the Nature of Hernia Congenita’.^28^ In 1755–6 John Hunter had examined the descent of the testis in the fetus and found a rare case in which the intestine and omentum lay within the tunica vaginalis of the testis. This led Hunter to investigate the natural descent of the testis from the abdominal cavity into the scrotum. John Hunter learnt of Albrecht von Haller’s (1708–1777) observations on hernia congenita from Haller's book *Opuscula Pathological*^29^ and described non-descent of the testis and the development of a sac, resulting in a hernia congenita or infantile hydrocele. At that time William Hunter described these findings to his students but was surprised in June or July 1756 to receive a letter from his then friend Percivall Pott with a copy of Pott's latest book A *Treatise on Ruptures*^30^ in which he came across a description of hernia congenita without credit being given either to himself or to his brother. In a later publication *An Account of a Particular Kind of Rupture Frequently Attendant upon Newborn Children,* Pott again presented his views on hernia congenita without mentioning either of the Hunter brothers.^31^

William Hunter wrote to Pott claiming that he, Pott, had been shown their work and ideas when he visited the Hunters’ anatomical school and had seen the preparations of how the intestine could descend into the hernial sac forming hernia congenita.

William Hunter wrote:

The [Potts’] treatise came out in the month of March 1757, it hardly contained one new idea. It was what any of my pupils might have written; (for the cases given in the end supported only an uncontested fact) and yet neither my brother's name nor mine was mentioned. It bore strong marks of second-hand observation and of a time serving hurry in the composition.^32^

Pott replied, defending himself eloquently and denying that he had even seen the Hunters’ description of hernia congenita, and stated that he did not seek this dispute. Pott stated that he would never write another word on the subject and concluded he had no wish to prevent the Hunters from enjoying their reputation or any honour which might arise from their labours on this or any other subject.^33^

Fenwick Beekman (1882–1962), in his analysis of this event, concluded that the Hunters lacked sufficient evidence for proof of plagiarism by Pott. Beekman contextualised the dispute by saying that although this case appeared unseemly it was not unknown in eighteenth-century England to make charges and counter charges which were commonly aired in the public press.^34^

Did Percivall Pott knowingly publish other people's ideas? He certainly was a renowned and respected surgeon who lectured and wrote extensively, and his ideas may well have been contemporaneous with others which somehow became fused with his own opinions.

Nevertheless, at some subsequent point in the history of the treatment of fractures, the names Sharp and Pott became uncoupled, and the former was forgotten. It is thus Pott's description of the importance of relaxing the muscles in order to reduce a fracture/dislocation of the ankle that has lasted to this day and his name is firmly associated with the treatment of these injuries.

This poses the questions to why William Sharp's name is not well known today, specifically in the management of these severe injuries, despite the fact that so much about his life is well documented. Why did William Sharp not pursue his novel idea and produce his own textbooks? We would argue that, unlike Pott, Sharp did not see himself as an educator and was more concerned with treating patients, pursuing his interests in boating and music, and caring for his extensive family.

## William Sharp’s Life Outside Medicine

Music was possibly Sharp's greatest love, a lifelong pleasure which started in childhood. Durham, like most cathedral cities was a centre of musical activity; his uncle James Heseltine (1692–1763) was the cathedral organist, and his father Thomas frequently organised concerts, even producing a paper ‘*Rules for Tuning an Organ or Harpsichord.*’^35^ Thomas ensured all his children had a musical education, could play and sing well, and were used to performing. The line between professional and amateur was blurred; Granville, for instance, is included in Doane's Musical Directory of 1794 as a tenor and member of the Madrigal Society. William, James and Granville and their sisters continued the family tradition of musical performances, usually of sacred music, and glee evenings^36^ in London, at William's house in Mincing Lane and later at 8 Old Jewry. This was a large house with plenty of space – ‘a noble hall at the entrance larger than your great room at Bamburgh, 50 × 30ft’ wrote James to John Sharp in January 1769, ‘and a grand painted staircase larger than that at St Bartholomew's’.^37^ William was involved with the Castle music society and played the clarinet and owned a chamber organ. All the family played musical instruments and sang and in the summer months the whole family went on the river with their instruments and continued their performances. They became well-known for their ‘water schemes’ as Elizabeth frequently referred to them in her diary.^38^ The Sharps were not unusual in this, the river being a facility for recreation as much as for transport. William's friend Johan Zoffany, painter of the family portrait, *‘*as was the fashion of the day, had his own sailing yacht and used to give concerts on board’.^39^ The *Apollo* depicted by Zoffany was one of several boats owned by the brothers William and James Sharp. Another, *The Union*, was extremely commodious; it had numerous beds, sofas, dining furniture, two water closets, a stove and kitchen equipment, presumably manufactured by James at his foundry in Southwark. So enamoured of life on the river were William and Catherine Sharp that they regarded The Union Yacht (which he had commissioned) as their floating home before their final move to Fulham.

William's brother James died in 1783 and from then the enthusiasm for musical boating parties appears to have waned and the boats were sold at Christies in September 1786.^40^ William had already bought Stourton House in Fulham, close to All Saints Church beside Putney Bridge, and he lived there from 1786 for the rest of his life, continuing with his medical practice. Hester Grant notes that ‘as a fashionable London doctor, William's annual salary measured in the thousands’.^41^

It would be impossible to assess the life of William Sharp, without reference to his siblings, such was their involvement in each other's lives. He was especially devoted to his sister Elizabeth who lived most of her life as a widow.^42^ Although he rented rooms in the Temple, Granville lived his entire life with either William or James and, when he resigned his administrative post at the Royal Ordnance in protest at the war with America, they together offered him financial support indefinitely - *‘*the happiness of being together is worth the expense’.^43^

By providing him with a home and income, William, who outlived James by many years, enabled Granville to be an independent political campaigner and key figure in the abolition movement. It is known from his record as an elected Common Councillor in the City of London that James's politics, like Granville's were ‘consistently on the radical side’, he was an active supporter of John Wilkes (1725–1797) and against the war with America.^44^ By supporting Granville financially, it is reasonable to infer that William held similar views. Certainly, among his books in Fulham, which he catalogued in 1806, were editions of Wilkes’ speeches and letters.^45^

In 1767, the same year in which he presented his paper on fractures to the Royal Society, William Sharp became involved, together with his brothers Granville and James, in the legal case of an escaped slave, Johnathan Strong. This marked the beginning of Granville's life-long involvement in the abolition movement and led ultimately to the landmark Mansfield ruling in the case of James Somerset in 1772.

In 1765, Johnathan Strong a slave whose master David Lisle a lawyer from Barbados, had beaten him almost to death, managed to get himself to William Sharp's surgery in Mincing Lane:

I could hardly walk or see my way where I was going. When I came to him (William) and he saw me in that condition, the gentleman take charity of me and gave me some stoff to wash my eyes with and some money to get myself a little necessaries till next day. The day after I come to the gentleman and he sent me into the hospital and I was in there four months and a half. All the while I was in the hospital the gentleman find me in clothes, shoes and stockings and when I come out he paid for my lodging and a money to find myself some necessaries till he get me into a place.^46^

Having restored his health, William found employment for Strong with Brown, an Apothecary, which lasted until his former master, Lisle spotted him and had him imprisoned in the Poultry Compter, a small prison at Poultry, Cheapside in the City of London. From here Strong sent word of his plight to Granville Sharp who came to the prison and insisted that Strong had committed no offence and could not be released to a third party, namely Lisle, and a Jamaican planter, James Kerr, who had paid Lisle £30 for Strong. The case was heard by the Lord Mayor in his role as magistrate. Granville Sharp used the principle of habeas corpus, and Strong was discharged. Granville was inspired to set about his life's great endeavour: to obtain a legal ruling against the holding of slaves. William had not only been involved in this case, but his pledge of financial support enabled Granville to continue his campaign. This culminated in 1772 with the landmark case of James Somerset which Granville regarded as a personal battle with the judge William Murray, (1705–1793) 1st Earl of Mansfield. He assembled a brilliant legal team who he bombarded with briefings. On 22 June 1772 a ruling could be delayed no longer and was delivered ex tempore by Mansfield in Westminster Hall to a Court packed with public and press:

The state of slavery is of such a nature that it is incapable of being introduced on any reasons, moral or political, but only positive law which preserves its force long after the reasons, occasion, and time itself from which it was created, is erased from memory. It is so odious that nothing can be suffered to support it but positive law. Whatever inconveniences, therefore, may follow from a decision, I cannot say this case is allowed or approved by the law of England, and therefore the black must be discharged.^47^

Strangely, Granville did not attend the Court that day but stayed at home, at William's house in Old Jewry, until Somerset himself came to bring the good news. The judgement marked a turning point in the abolition campaign.

If William Sharp's politics were, in the language of the time, radical, like those of both his brothers James and Granville, they were decidedly not republican. The brothers might even be said to have been on cordial terms with the monarch. George III not only appreciated their musical performances and took tea on their boat but he also consulted James personally on detailed aspects of canal building and consulted William professionally. In 1783 William received a note from George III asking him to arrange for the surgeon Sir Richard Jebb (1729–1787) to call on him at Windsor.^48^ It is curious that the King did not send for Jebb directly, and this suggests a degree of familiarity with William. Sometime later in February 1799 in a letter to his sister Judith in Durham, William reported that the Queen had commanded him to attend the young Princess Amelia (1783–1819) for a problem with her knee. By this time Sharp had already been semi-retired for a decade.^49^ Although without any formal royal appointment, William Sharp's association with Royalty would undoubtedly have enhanced his reputation in society and increased the demand for his surgical services. It certainly gave him the confidence, when sitting for Zoffany, to wear the Windsor uniform, a badge of loyalty to the Crown.

**Figure 4.**
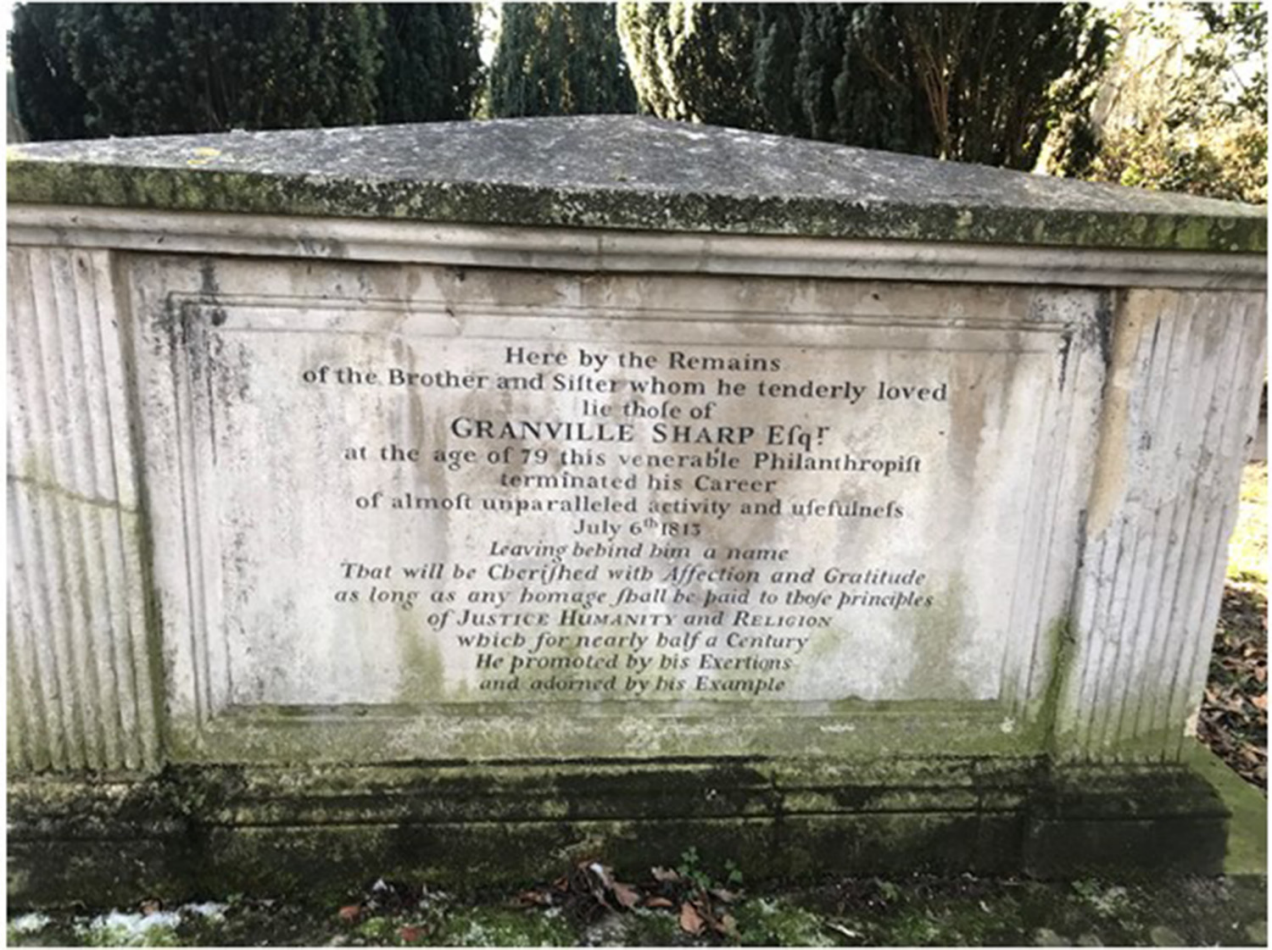
Sharp family tomb, All Saints Church, Fulham London, where William, Elizabeth Prowse, Granville and Catherine were buried.

On 17 March 1810 William Sharp died and was buried at All Saints Church Fulham, beside his sister Elizabeth Prowse who had died a few weeks earlier. Granville continued living in William’s house in Fulham until his death on 15 February 1813. He was buried in the same tomb, his coffin having been carried by a group of men which included Thomas Clarkson and William Wilberforce (Figure 4).

A year later in 1814 William's wife Catherine was also buried in the tomb. Their only child, Mary predeceased her in 1812, leaving a son aged five years. Mary had married into the Lloyd-Baker family of Gloucestershire and was the only one of the Sharp family to produce descendants, still alive today. 

## Conclusion

We argue that William Sharp made a seminal contribution to the treatment of fracture/dislocations of the ankle when he introduced the concept of relaxing the knee joint in order to allow the dislocation to be reduced. However, although a contemporary of Pott and on the staff of the same hospital, he remains, in the history of surgery, a largely forgotten figure. It is unfortunately not clear whether Sharp was simply describing a method that was used at St Bartholomew's Hospital at the time or whether he was suggesting this technique as a revolutionary advance in the management of these severe injuries. What is evident is that Sharp delivered his paper to the Royal Society of London in 1767 describing how to successfully reduce fracture/dislocations of the ankle, which was then later described by Percivall Pott in his book in 1769. This would suggest that Sharp was the first to describe the appropriate method for treatment of what has become known as Pott's method for treating fracture dislocations of the ankle.

Pott was also fortunate in having a biographer in the family, James Earle his son in law, a former pupil, and a surgeon.

James Earle (1755–1817) wrote

In 1768 he produced A new edition of his book on the Injuries to which the head is liable from External Violence, accompanied with what is entitled A Few General Remarks, but which is really a complete system on fractures and dislocations. … The novelty of the doctrine contained in this treatise relates principally to the position of the injured limb. On its publication it met with some resistance but has now subdued the first prejudices; and I believe I may venture to say, is become almost the universal practice.^50^

The problem, with William Sharp, however, is that in his lifetime he only wrote one letter published in the Proceedings of the Royal Society and one drawing of a stone removed from the bladder of Reverend Mr T-C.^51^ Rather than writing, he seems to have preferred to treat his patients, play music, and go boating on the Thames with his devoted family.

It seems plausible also to suggest that any inclination William might have had to write about his work, once he had retired, could have been constrained by problems with his failing eyesight, vividly described by Granville in 1787.^52^ It is also conceivable that he was aware of Pott's confrontation with the Hunters some ten years previously and had no wish to become embroiled in a battle with his surgical colleague on who had made the original observation.

Also, although William’s friend Prince Hoare (1755–1834), the English painter and dramatist,^53^ was commissioned by Catherine Sharp to write a biography of her younger brother, Granville, no such record was made of William's life. His eulogy was given at All Saints Church by the Reverend John Owen, founder of the British and Foreign Bible Society.^54^

History indeed can be unkind to those actors who do not establish themselves by contemporaneous methods of communication and in William Sharp's case this would have been the writing of a textbook. We have put forward a variety of explanations for this, namely his lifelong commitment to music, his love of boating, his many family commitments, a relaxed and secure personality type, no financial imperative and evidently no interest in self-promotion.

It would seem therefore that William Sharp, chose not to publicise his seminal observations on the treatment of severe injuries of the ankle and chose his memorial when he commissioned Johan Zoffany to paint him, wearing the Windsor livery, on board his yacht, with the family he loved, a happy band of amateur musicians. In the background is All Saints Church in Fulham where William Sharp lies buried with his wife, his sister Elizabeth and brother Granville. William stands at the back, hand on the tiller, waving his hat with a look of quiet contentment. This is his legacy.
